# Metabolic system alterations in pancreatic cancer patient serum: potential for early detection

**DOI:** 10.1186/1471-2407-13-416

**Published:** 2013-09-12

**Authors:** Shawn A Ritchie, Hirofumi Akita, Ichiro Takemasa, Hidetoshi Eguchi, Elodie Pastural, Hiroaki Nagano, Morito Monden, Yuichiro Doki, Masaki Mori, Wei Jin, Tolulope T Sajobi, Dushmanthi Jayasinghe, Bassirou Chitou, Yasuyo Yamazaki, Thayer White, Dayan B Goodenowe

**Affiliations:** 1 Phenomenome Discoveries, Inc., Saskatoon, SK, Canada; 2 Department of Surgery, Osaka University Graduate School of Medicine, Osaka, Japan; 3 Current address: Pan-Provincial Vaccine Enterprise Inc. (PREVENT), Saskatoon, SK, Canada; 4 Current address: Department of Community Health Sciences, Hotchkiss Brain Institute Clinical Research Unit & Institute for Public Health, University of Calgary, Calgary, AB, Canada; 5 Glycozym, Inc., Beverly, MA, USA

**Keywords:** Pancreatic cancer, Biomarker, Metabolism, Metabolomics, Screening, Early detection, Mass spectrometry

## Abstract

**Background:**

The prognosis of pancreatic cancer (PC) is one of the poorest among all cancers, due largely to the lack of methods for screening and early detection. New biomarkers for identifying high-risk or early-stage subjects could significantly impact PC mortality. The goal of this study was to find metabolic biomarkers associated with PC by using a comprehensive metabolomics technology to compare serum profiles of PC patients to healthy control subjects.

**Methods:**

A non-targeted metabolomics approach based on high-resolution, flow-injection Fourier transform ion cyclotron resonance mass spectrometry (FI-FTICR-MS) was used to generate comprehensive metabolomic profiles containing 2478 accurate mass measurements from the serum of Japanese PC patients (*n*=40) and disease-free subjects (*n*=50). Targeted flow-injection tandem mass spectrometry (FI-MS/MS) assays for specific metabolic systems were developed and used to validate the FI-FTICR-MS results. A FI-MS/MS assay for the most discriminating metabolite discovered by FI-FTICR-MS (PC-594) was further validated in two USA Caucasian populations; one comprised 14 PCs, six intraductal papillary mucinous neoplasims (IPMN) and 40 controls, and a second comprised 1000 reference subjects aged 30 to 80, which was used to create a distribution of PC-594 levels among the general population.

**Results:**

FI-FTICR-MS metabolomic analysis showed significant reductions in the serum levels of metabolites belonging to five systems in PC patients compared to controls (all *p*<0.000025). The metabolic systems included 36-carbon ultra long-chain fatty acids, multiple choline-related systems including phosphatidylcholines, lysophosphatidylcholines and sphingomyelins, as well as vinyl ether-containing plasmalogen ethanolamines. ROC-AUCs based on FI-MS/MS of selected markers from each system ranged between 0.93 ±0.03 and 0.97 ±0.02. No significant correlations between any of the systems and disease-stage, gender, or treatment were observed. Biomarker PC-594 (an ultra long-chain fatty acid), was further validated using an independently-collected US Caucasian population (blinded analysis, *n*=60, *p*=9.9E-14, AUC=0.97 ±0.02). PC-594 levels across 1000 reference subjects showed an inverse correlation with age, resulting in a drop in the AUC from 0.99 ±0.01 to 0.90 ±0.02 for subjects aged 30 to 80, respectively. A PC-594 test positivity rate of 5.0% in low-risk reference subjects resulted in a PC sensitivity of 87% and a significant improvement in net clinical benefit based on decision curve analysis.

**Conclusions:**

The serum metabolome of PC patients is significantly altered. The utility of serum metabolite biomarkers, particularly PC-594, for identifying subjects with elevated risk of PC should be further investigated.

## Background

Pancreatic Cancer (PC) is one of the most challenging cancers to detect and treat. Currently, PC is diagnosed by imaging methods such as endoscopic ultrasonography (EUS) or helical CT scan, typically only after the presentation of symptoms serious enough to warrant the procedure [1,2]. The low incidence of PC combined with the invasiveness and cost of endoscopic-based approaches make them unsuitable for average-risk population screening. Accordingly, over 80% of PC cases are detected at advanced stages of the disease, wherein the five-year survival rate is less than 3% [3]. A non-invasive screening test that could identify high PC-risk subjects for whom the benefit of endoscopic examination would outweigh the risk of the procedure is needed, analogous to serum-based GTA-446 testing to identify high risk colorectal cancer (CRC) subjects who should undergo colonoscopy [4].

The primary risk factors for PC are similar to those for other cancers, and include age, diet, obesity, exercise status, smoking status, gender, diabetes, family history and geography (see [5-9] for review). With respect to PC specifically, chronic pancreatitis may also be a risk factor [10]. It is likely that many of these factors contribute cumulatively to risk over years, given that PC (or any cancer) does not spontaneously appear within the body. Since the pancreas is intricately involved in metabolism, and since most of the aforementioned risk factors have a strong metabolic component, we questioned the possibility of a unique metabolic signature correlating with PC.

Non-targeted metabolomics is a hypothesis-generating approach aimed at broadly characterizing the metabolic composition of a sample in an unbiased manner by detecting and identifying as many components in a sample as possible [11-13]. In this study, a combination of high-resolution, flow-injection Fourier transform ion cyclotron resonance mass spectrometry (FI-FTICR-MS) and flow-injection tandem mass spectrometry (FI-MS/MS) was used to identify and confirm specific dysregulated metabolic systems associated with PC in two ethnically and geographically diverse populations.

## Methods

### Study cohort

All blood samples in this study were collected under fasted conditions and serum prepared off the clot using red-topped vacutainer tubes. All samples were stored at −80°C until analysis. Discovery samples from Osaka Medical University, Japan, were collected between 2005 and 2007, and included 40 PC patients and 50 matched disease-free control subjects. The study was approved by the Osaka University Graduate School of Medicine Medical Ethics Board, ethics board number 213, and all subjects signed informed consents. Samples were drawn, processed and stored in a consistent manner by qualified physicians. Of the 40 PC patients, 24 were drawn at the time of surgery immediately following anesthetization, and 16 were drawn prior to surgery (not under anesthesia). Of the total 40, 20 were collected prior to any chemo or radiation therapy, and 20 were collected during or after at least one cycle of treatment. Of the 24 drawn at time of surgery, 13 had undergone treatment. Of the 16 not collected at surgery, 7 had undergone treatment. Detailed pathology reports were collected on all subjects. Further information is provided in Table 1. Disease-free Japanese control subjects were recruited on the basis that they had no history of cancer and that serum levels of the tumor markers CEA, CA19.9, SCC, AFP, CA125, PSA and CA15.3 were negative.

**Table 1:** Description of populations used in the study

**Japanese controls**	
All (*n*)	50
Female (*n*)	20
Male (*n*)	30
Age (years, range)	63.8, 40-75
**Japanese pancreatic cancer**	
All (*n*)	40
Stage I (*n*)	4
Stage II (*n*)	4
Stage III (*n*)	5
Stage IVa (*n*)	16
Stage IVb (*n*)	11
Collected at surgery^1^ (*n*)	24
Not collected at surgery (*n*)	16
Sample collected after treatment^2^ (*n*)	20
Sample collected prior to treatment (*n*)	20
Female (*n*)	14
Male (*n*)	26
Average age (years, range)	65.2, 31-79
**USA Caucasian control 1**	
All ages (*n*)	1000
30-39 yrs (*n*)	103
40-49 yrs (*n*)	280
50-59 yrs (*n*)	201
60-69 yrs (*n*)	214
70-80 yrs (*n*)	202
Female (*n*)	598
Male (*n*)	402
**USA Caucasian control 2**	
All (*n*)	40
Female (*n*)	8
Male (*n*)	21
Gender unknown (*n*)	11
Average age (years, range)	42.7, 18-60
**USA Caucasian pancreatic cancer**	
All (*n*)	14
Female (*n*)	3
Gender unknown (*n*)	11
Average age (years, range)	70.4, 57-85
**USA Caucasian IPMN**	
All, gender unknown (*n*)	6
Average age (years, range)	73.0, 61-80

^1^Samples collected under anesthesia.

^2^Chemo/radiation therapy (at least one cycle).

The North American validation samples were provided by the Cooperative Human Tissue Network (CHTN), which is funded by the National Cancer Institute. The samples included serum from 14 Caucasian PC adenocarcinoma patients, six patients with indraductal papillary mucinous neoplasms (IPMN), and 40 Caucasian cancer-free controls with no history of cancer. Based on the discovery results, the study was powered to a sensitivity and specificity of 88% at 10% precision according to the method of Malhotra *et al.*[14], resulting in a confidence level of 88%. The study was approved by Institutional Review Board #4 of the University of Pennsylvania and all patients signed informed consents. Following analysis, results were sent to Glycozym Inc. for statistical analysis and un-blinding.

To determine the distribution of PC-594 in the general, average-risk population, 1000 anonymous (depersonalized) reference serum samples (598 females and 402 males) were randomly selected from routine clinical blood draws at the Central Ohio Primary Care lab (USA control 1, Table 1). The population included at least 100 samples for each decade of life between age 30 and 80.

### Sample extraction

All serum samples were stored at −80°C until thawed for analysis, and were only thawed once. All extractions were performed on ice. Serum samples were prepared for FI-FTICR-MS by sequential extractions with 1:1:5 volumes of 1% ammonium hydroxide and ethyl acetate (EtOAc) three times. Samples were centrifuged between extractions at 4°C for 10 min at 3500 rpm, the organic layer removed, and transferred to a new tube (extract A). After the third EtOAc extraction, 0.33% formic acid was added, followed by two more EtOAc extractions. Following the final organic extraction, the remaining aqueous component was further extracted twice with water, and protein removed by precipitation with 3:1 acetonitrile (extract B). A 1:5 ratio of EtOAc to butanol (BuOH) was then evaporated under nitrogen to the original BuOH starting volume (extract C). All extracts were stored at −80°C until FI-FTICR-MS analysis.

### FI-FTICR-MS analysis

All analyses were performed on a Bruker Daltonics APEX III Fourier transform ion cyclotron resonance mass spectrometer equipped with a 7.0 T actively shielded superconducting magnet (Bruker Daltonics, Billerica, MA). Extracts B and C were diluted in methanol:0.1% (v/v) formic acid and analyzed by electrospray ionization (ESI) in the positive mode, and methanol:0.1% (v/v) ammonium hydroxide in the negative mode. Undiluted extract A was analyzed by flow injection using atmospheric pressure chemical ionization (APCI). The flow rate for all analyses was 600 μL/hr. Details of instrument tuning and calibration conditions have been previously reported [15]. All spectra were calibrated to a mass accuracy of <1 PPM relative to the theoretical masses of internal standards. Sample peak intensities were aligned and visualized as a two-dimensional array using DISCOVAmetrics^TM^ 4.0 (Phenomenome Discoveries Inc.).

### FI-MS/MS analyses

FI-MS/MS analyses were performed as previously described with modifications [16]. All analyses were performed on a triple quadrupole mass spectrometer (API 4000, Applied Biosystems) coupled with an Agilent 1200 LC system. Methods were based on multiple reaction monitoring (MRM) of parent/fragment ion transitions specific for each metabolite (see Additional file 1, Tables S1, S2, S3, S4, S5 and S6). The mobile phase flow rate for all methods was 600 μL/min. Instrument linearity was determined by the serial dilution of standard in the appropriate extract of Randox serum (Human Serum Precision Control Level II). All samples were analyzed in a randomized blinded manner and were bracketed by known serum standard dilutions. Results were based on ratios of integrated analyte peak area to the appropriate internal standard.

Panel-specific conditions and parameters were as follows: For the PtdCho and SM panels, 12 μL of extract B was mixed with 108 μL mobile phase and 15 μL of 0.5ug/ml PtdCho16:0(D31)/18:1 as an internal standard. For the lysoPC panel, 12 μL of extract B was mixed with 108 μL mobile phase and 15 μL of 0.5 ug/ml of lysoPC18:0(D35) as the standard. All deuterated standards were purchased from Avanti Polar Lipids. The mobile phase for the cholines consisted of a 3:1 ratio of acetonitrile to 1% formic acid in ddH_2_O. 60 μL of sample cocktail (50 μL for sphingomyelins), were injected by flow injection analysis (FIA) and monitored under negative ESI using the parent-daughter ion transitions as listed in Additional file 1. Parameters for the PlsEtn and PtdEtn panels have been previously reported [16]. Long-chain fatty acids were analyzed using negative atmospheric pressure chemical ionization (APCI) as previously described [15].

The quantitative PC-594 FI-MS/MS method was developed on an Ionics 3Q triple-quadrupole mass spectrometer coupled to an Agilent 1200 LC system as above. The Q1/Q2 MRM transitions monitored were 593/557 in APCI negative mode using extract A and ^13^C-cholic acid as a standard [4]. The isocratic mobile phase was comprised of water-saturated ethyl acetate run at a flow-rate of 350 μL/min. The auto-sampler temperature was 22°C and column oven 35°C. Sample injection volume was 100 μL with a draw speed of 200 μL/min with a 400 μL/min injection speed using an APCI source in negative mode. The pause time was 5 ms, scan speed was 0.56 sec/scan, and corona discharge, -4. MS system temperatures were: drying gas, 100°C; HSID, 200°C; nebulizer gas, 350°C; and probe, 350°C. Concentration of PC-594 was determined by extrapolation using a ^13^C-cholic acid standard curve and was reported as ^13^C-cholic acid equivalents (CAEs). Acceptance criteria were that pooled reference sample reproducibility was <15% RSD and standard curve R-squared values was >0.98.

### Statistics

Metabolite array generation and hierarchical clustering were performed using DISCOVAmetrics™ software (Phenomenome Discoveries Inc., Saskatoon). Two-tailed unpaired Student’s t-tests were used to compare PC and control samples for all masses. False-discovery rate (FDR) was controlled for by the method of Benjamini-Hochberg [17]. Principal components analysis (PCA) was performed in STATA. PCA factor loadings, score plots, uniqueness and R2 correlations are shown in Additional file 2. We performed both the Bartlett’s test for non-zero correlation and the Kaiser-Meyer-Olkin (KMO) test for sample adequacy prior to PCA. We then selected masses for which the *p*-value for the Bartlett’s test was less than .05 and the KMO was greater or equal to 0.81. Random forest (RF) classification, a non-parametric classification technique that utilizes a classification and regression tree method for prediction and variable selection, was used to identify the most discriminatory accurate masses between PC cases and control subjects. The dataset was split into two-thirds (n = 58; 22 PC and 36 control) for training and one-third (n = 32; 18 PC and 14 control) for testing. The variable selection technique of RF was used to rank order the masses according to their contribution to the accuracy of the classifier and by the mean decrease in Gini index. RF analysis was conducted in R.2.15.1 (http://cran.r-project.org/), and the ROC curve for the RF classifier was generated using JROCFIT 1.0.2 based on outputted cancer positive probabilities. FI-MS/MS data analysis was carried out using Analyst 1.5, Microsoft Office Excel 2010, and STATA 12. ROCs were based on the continuous distribution of tandem-MS results. Beeswarm jitter plots in were performed using R.2.15.1.

Calibration was performed to evaluate agreement between observed and predicted probabilities based on the Random Forest probability outputs of the Japanese patients and PC-594 levels in USA patients. For the Random Forest prediction model (calibration plot shown in Additional file 3), a PC probability of 25% was used as the cutoff; for the calibration plots of the USA data (shown in Additional file 4), cut-offs yielding 5% positivity in the control groups were used. The difference between a perfect model (represented by the first diagonal) and the predicted models based on logistic regression were represented by the average error (E_avg_) and the maximum errors (E_max_). All computations and curves were performed using STATA 12.

To assess the clinical benefit of our models, we performed decision curve analysis (DCA) according the method of Vickers [18,19]. The goal was to determine whether PC-594 screening prior to performing endoscopic ultrasound (EUS) would offer any clinical benefit over either performing, or not performing, EUS on everyone. The approach models (and compares) the clinical benefits at increasing probability thresholds (*p*_*t*_’s) for the above scenarios. DCA was performed on the USA PC population (and USA 2 controls) using the DCA package in STATA 12. The net clinical benefit of 0.14, at a 20% *p*_*t*_ was calculated as the difference in benefit between screen all (~0.08) and the model based on PC-594 (~0.22).

## Results

### FI-FTICR-MS metabolomic analysis

Serum samples from 40 PC patients and 50 controls (Table 1) were extracted and analyzed by FI-FTICR-MS as described in the methods, resulting in a two-dimensional metabolite array containing 2478 sample-specific accurate masses. We used three independent statistical methods to investigate the data. First, we reduced the dimensionality of the data using principal components analysis (PCA) to determine whether variance in the data correlated with the presence of PC. Second, we performed hierarchical clustering (HCA) using a Pearson distance metric to group masses belonging to related metabolic systems. Third, we used Random Forest (RF) for its classification and built-in cross-validation capabilities, and to identify masses with the strongest discriminating ability.

**Figure 1:**
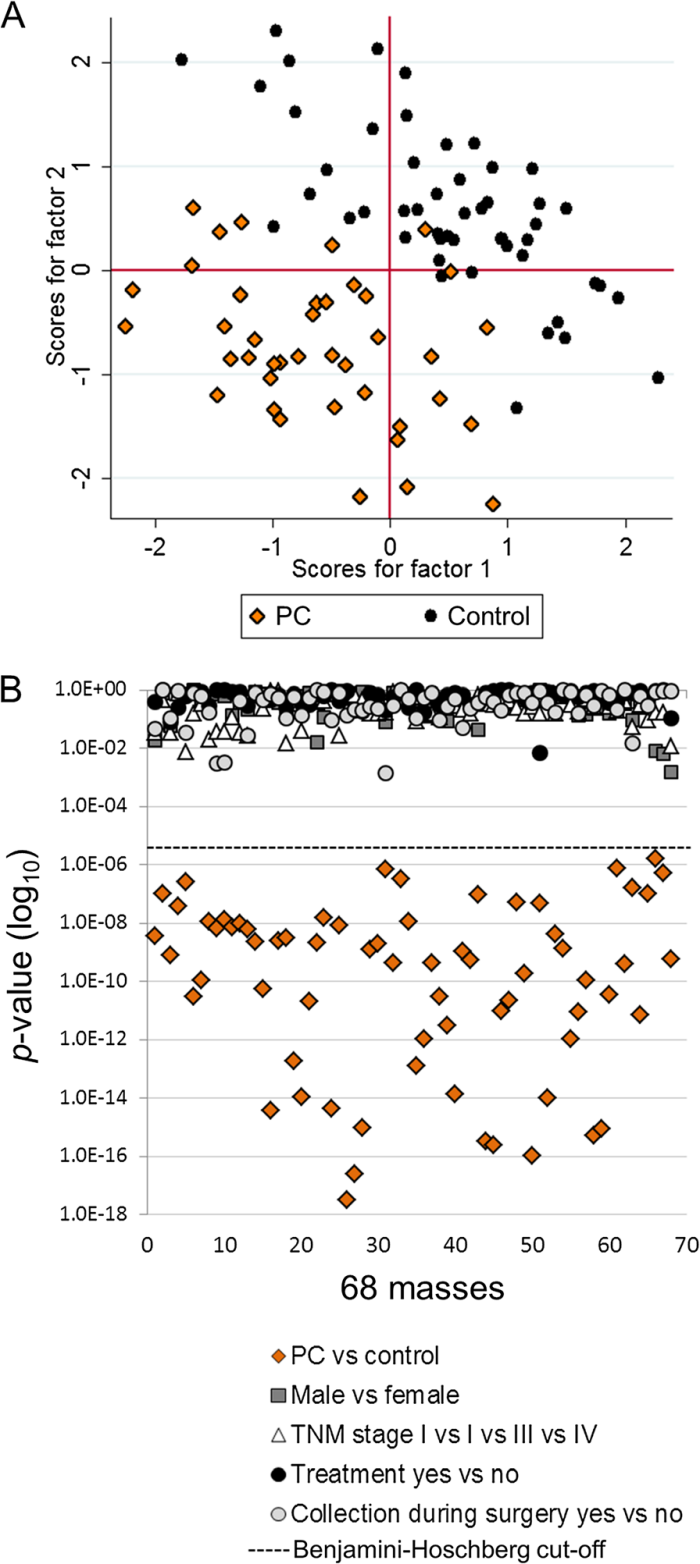
**Principal components analysis (PCA). A**, PCA based on 68 masses selected following Benjamini-Hoshberg FDR correction (*p*<2.5E-5), Bartlett’s test for sphericity (*p*<0.05), and a KMO greater than 0.81. Each point represents a patient profile, colored by disease state (black circles, control subjects; orange diamonds, PC patients). **B**, Scatter plots of the *p*-values (log_10_) for each of the 68 masses based on t-test comparisons between clinical variables (see legend). See Additional file 2 for PCA parameters and *p*-values.

We first computed the *p*-value of each mass between PC patients and controls, controlling for false-discovery rate (FDR) using the method of Benjamini-Hochberg [17]. Prior to performing principal components analysis (PCA), we performed the Bartlett’s test for non-zero correlation [20] and the Kaiser-Meyer-Olkin (KMO) test for sample adequacy [21], and accepted only masses for which the *p*-value of the Bartlett’s test was less than 0.05 and the KMO was greater than or equal to 0.81 (0.80 and above is considered meritorious). This approach identified 68 masses that were then subjected to PCA analysis (Figure 1A). Only components for which the eigenvalue was greater or equal to 1 were retained. The PCA plot showed separation between PC patients and controls orthogonally along PC1 and PC2. The cumulative variance for factors one and two was 63%, and the average square multiple correlation (R2) among the 68 masses was 0.96. The PCA was highly significant with a *p*-value < 0.00001 for the likelihood ratio (LR) test (independent versus saturated model). The factor variances, loadings, uniqueness, square factor loadings (Q2) and R2 for each mass are shown in Additional file 2. The results suggested the presence of biochemical differences between the sera of PC patients and controls.

We investigated potential bias from other clinical variables by calculating the *p*-values for each of the 68 masses according to gender, disease stage, whether patients had undergone treatment prior to sample collection, and whether the sample collection was taken at time of surgery. None of the *p*-values for any of the comparisons were significant (FDR considered), and there were several orders of magnitude in the difference between the *p*-values for disease status (PC versus control) compared to all other variables including gender, disease stage, treatment status, and sample collection time relative to surgery (Figure 1B). The results confirmed that the correlations were specific to PC. All *p*-values are listed in Additional file 2.

**Figure 2:**
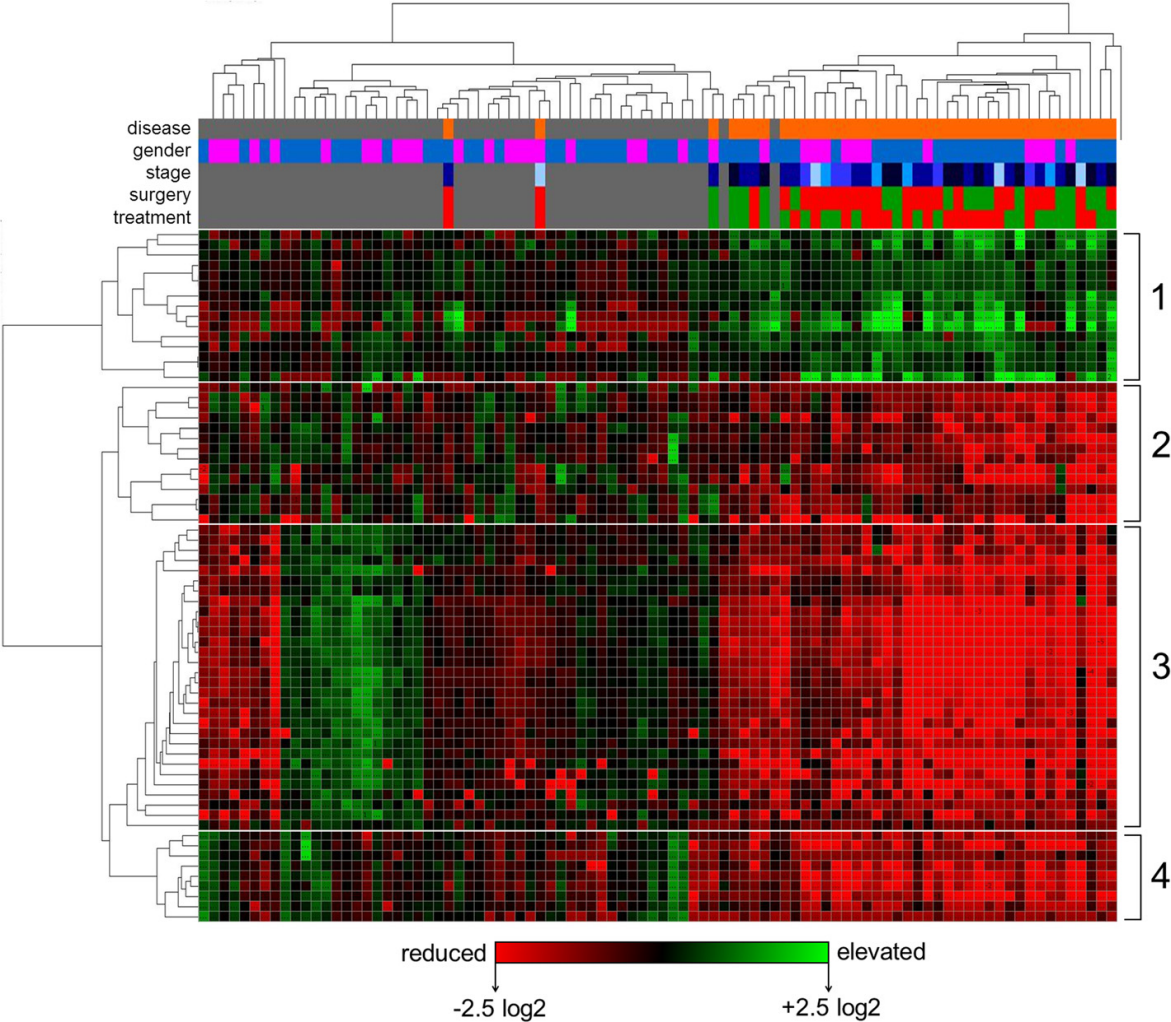
**Hierarchically-clustered metabolite array.** The same dataset used for PCA in Figure 1 was log_2_ normalized to the control mean and hierarchically clustered by mass (using a Pearson correlation) and by subject (using a Manhattan correlation). Colored rows at the top of the array indicate variable assignments for subjects (disease status, grey = control subject, orange = PC patient; gender, blue = male, pink = female; stage, light blue = stage I, dark blue = stage IV; surgery, red = yes, green = no; chemo/radiation treatment, red = yes, green = no). The heatmap is colored according to log_2_ intensity ratio; red = lower relative to control mean, green = higher relative to control mean. Metabolite clusters are numbered one through four on the right.

To identify metabolic relationships among the 68 masses selected above, we performed hierarchical clustering (HCA) by mass using a Pearson distance metric, and by sample using a Manhattan distance metric (Figure 2). Intensities were control mean-normalized (log_2_). PC patient and control samples split into two separate clusters, with only four subjects (two PC and two controls) misclassified (top dendrogram). No clusters correlating with gender, stage, treatment or surgery were observed (see variable header, Figure 2).

Hierarchical clustering of masses by Pearson correlation resulted in four primary clusters (Figure 2, see left dendrogram). This approach groups masses based on their similarity of intensity between samples, meaning that masses with related fold-changes between subjects cluster together, independent of their absolute levels. Often, metabolites belonging to the same system show a similar pattern, which makes this approach particularly useful for quickly grouping non-targeted discovery data into specific metabolic families, for identifying isotope and adducts, and for aiding in molecular identification. Overall, cluster one represented masses with mean elevated levels among PC patients, while clusters two through four represented masses with reduced levels (Figure 2).

**Figure 3:**
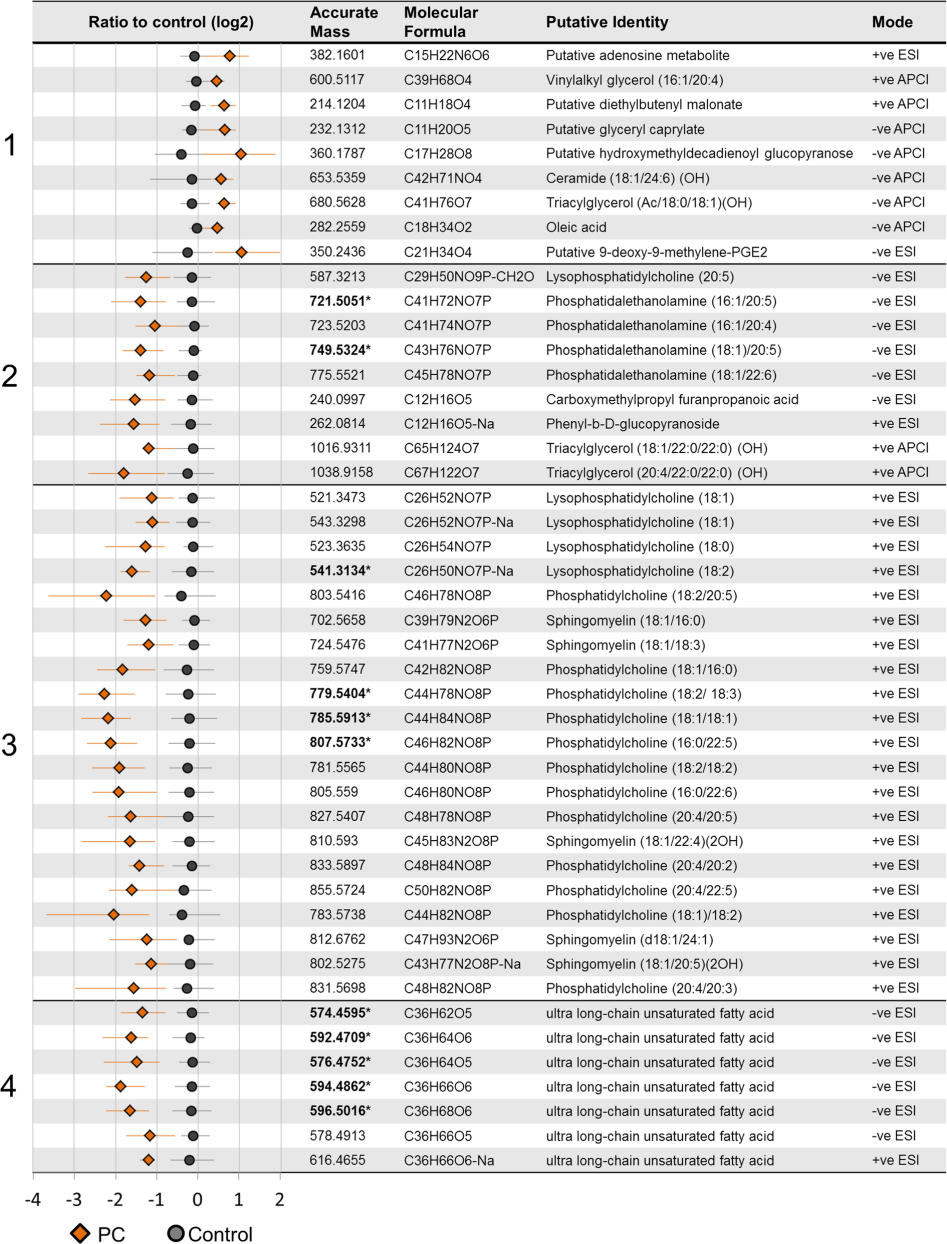
**Putative assignments of selected **^**12**^**C metabolites from HCA clusters in Figure** 2**.** The corresponding cluster numbers from Figure 2 and the mean log_2_ ratios relative to control for each metabolite are shown along the left side. Orange diamonds = PC patients, grey circles = control subjects. The horizontal whiskers represent the 25^th^ to 75^th^ percentiles of the control-normalized ratios. Adjacent columns in order from left to right indicate the detected accurate masses, the computationally predicted molecular formulas, the putative identities, and the mass spectrometry source modes. Cluster **1** represented a diverse group of metabolites increased in PC patients relative to controls, while clusters **2**, **3** and **4** represented reductions in plasmalogen ethanolamines (PlsEtns), phosphocholine-containing metabolites (including PtdCho, lysoPC and SMs), and ultra long-chain fatty acids, respectively. Predicted side-chain speciations in the case of glycerolipids are shown in brackets. Bolded masses with asterisks indicate those selected by Random Forest classification.

Putative identifications were computationally assigned to most of the masses using a combination of accurate mass database searching (DISCOVAmetrics^TM^, Phenomenome Discoveries Inc.), statistical similarity clustering (based on Pearson correlation), online databases (such as Chemspider and SciFinder), and *de novo* computational molecular formula calculations. The control-normalized ratios (log_2_) for PC patients and controls, detected accurate masses, predicted molecular formulas, putative identities and the MS detection modes of selected ^12^C masses from each cluster in Figure 2 are shown in Figure 3.

Cluster one contained a diverse group of elevated metabolites including several predicted shorter-chain organic molecules (containing 11 to 21 carbons), a predicted triacylgycerol, and a putative adenosine-related metabolite. Clusters two, three and four, with lower intensities in PC patients relative to controls, were represented by several classes of glycerophospholipids and ultra long-chain fatty acids. Specifically, cluster two contained several PlsEtns (PtdEtns containing a vinyl-ether linkage at the SN1 position), while cluster three contained multiple phosphocholine-related systems including PtdChos, lysoPCs, and several SMs. Cluster four comprised novel ultra long-chain hydroxylated fatty acids (LCFAs) containing 36 carbons and five or six oxygen that we characterized in previous studies [15]. Before confirming these identities by tandem MS, we employed Random Forest (RF), a cross-validation classification approach, to identify the most predictive markers in the dataset (below).

### Random forest classification

We used Random Forest (RF) to build a classification model and to identify masses with the most discriminating potential. RF is a statistical classification method based on an ensemble or multiple decision tree approach that incorporates built-in cross-validation during the training phase. The intrinsic cross-validation is performed by constructing trees using different bootstrap sample groups of approximately one third the original data [22].

**Figure 4:**
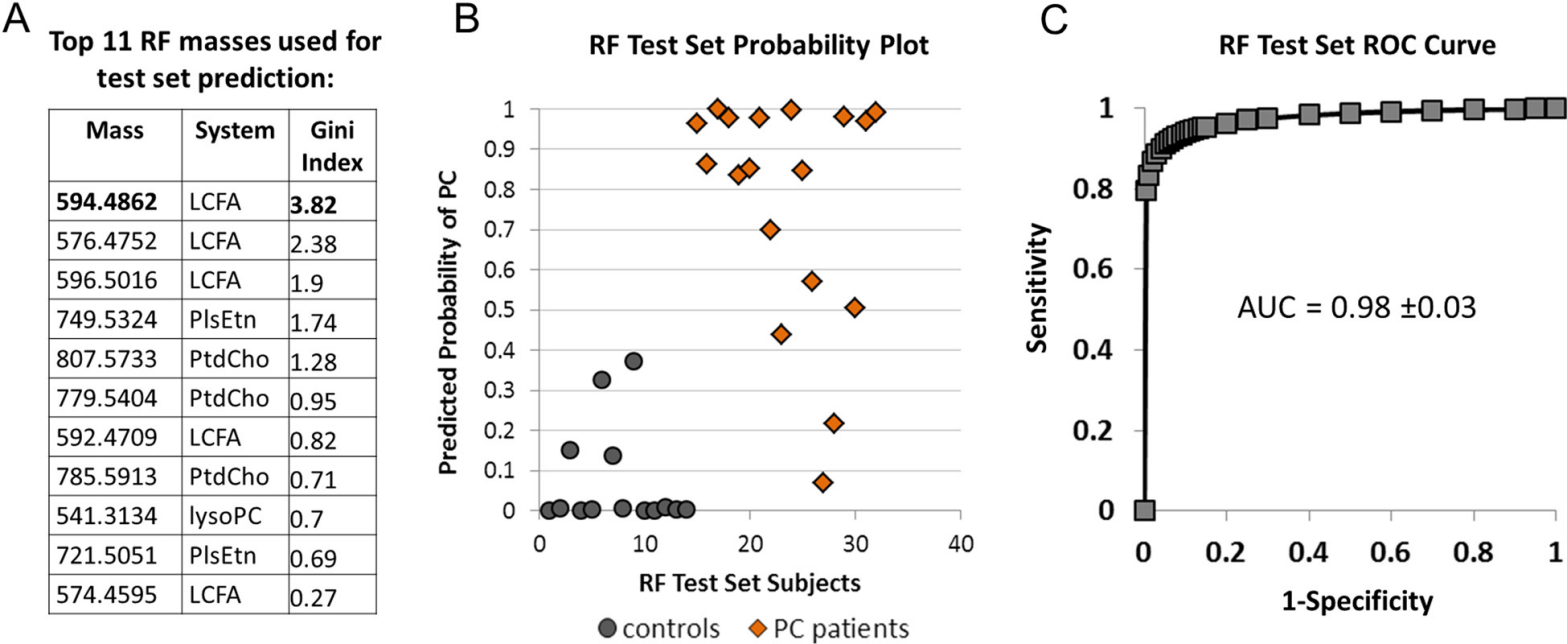
**Random Forest (RF) classification.** An RF classifier based on two-thirds of the sample population (n=58) was first created to rank the top 300 masses differentiating controls from PC patients (all *p*<0.001, FDR corrected) based on their contribution to the classification accuracy and the Gini index (See Additional file 3). The top 11 masses **(A)** based on these criteria were selected and a classifier created to predict the identity of the blinded test set samples as shown in **B**. **C**, ROC curve (with AUC ±95% CI) based on the RF predicted PC probabilities for each of the 32 blinded samples.

We split the discovery dataset into two-thirds (*n* = 58; 22 PC and 36 control) for training and one-third (*n* = 32; 18 PC and 14 control) for testing. We created the training classifier first using the 300 most significant masses (based on *p*-value) between PC patients and controls. The masses were then ranked based on percent contribution to the classifier accuracy and the mean decrease in Gini index (Additional file 3). The top 20 masses based on both the contribution to classifier accuracy as well as the mean decrease in Gini index were compared and reduced to a common 11 (Figure 4A). A second RF classifier based on these 11 masses was then applied to the blinded test set, which correctly classified 29 of the 32 samples (90.6%). The predicted probability of each test set sample as PC is shown in Figure 4B. The ROC curve based on the probabilities resulted in an AUC of 0.98 (95% CI, 0.95-1.0, Figure 4C). Calibration (see [23] for review) between the predicted and actual probabilities using a logistic regression approach (see Methods) showed a maximum difference (E_max_) of 0.12 and an average difference (E_avg_) of 0.06, indicating reliability in the prediction model (See Additional file 3). When the masses were ranked according to their contribution to the classifier, mass 594.4862 (Da) was the most critical according to the Gini index (Figure 4A). The results confirmed that the predicted metabolic systems, and in particular mass 594.4862, were highly associated with PC.

### FI-MS/MS verification

We confirmed the identity of the metabolic systems predicted above, including side-chain speciations of glycerophospholipids, by designing flow-injection tandem MS (FI-MS/MS) assays for each system (see Methods). Representative CID patterns and associated extracted ion currents (EICs) of metabolites for each of the metabolic systems, as observed in PC patients and controls, are shown in Additional file 5. Comprehensive lists of the parent-daughter ion transitions for each metabolite of each panel are shown in Additional file 1.

**Figure 5:**
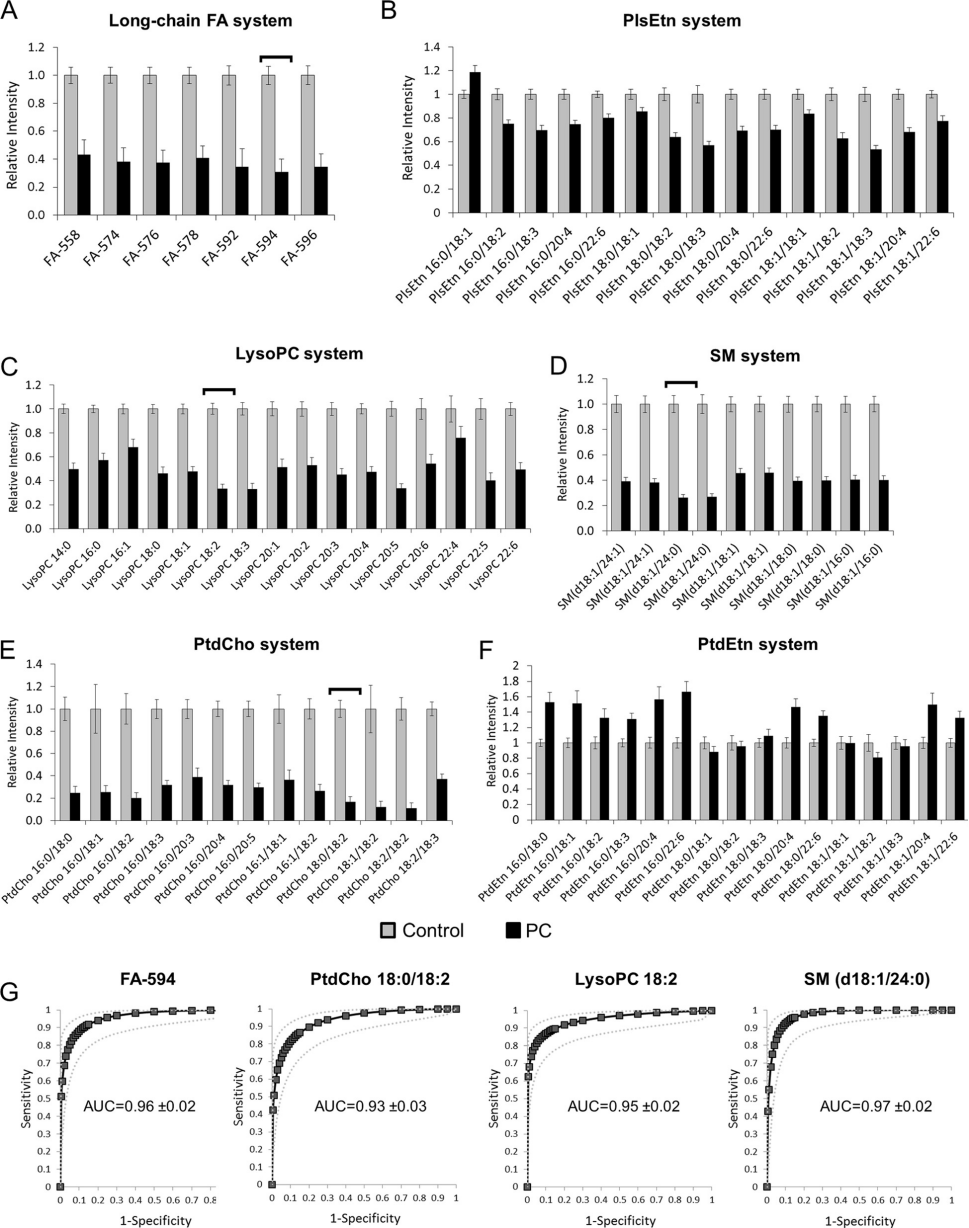
**Relative levels of metabolites belonging to six different metabolic systems based on FI-MS/MS analysis. A**, 36-carbon long-chain FA system; **B**, PlsEtn system; **C**, lysoPC system; **D**, SM system; **E**, PtdCho system; **F**, PtdEtn system. Error bars represent ±1 SEM. **G**, ROC curves based on the metabolites from four of the metabolic systems with the lowest *p*-values (shown by the horizontal brackets in **A**, **C**, **D** and **E**). AUCs are shown with 95% CI. See Additional file 1 for parent-daughter transitions.

The results of the FI-MS/MS analysis for each metabolic system were consistent with the FI-FTICR-MS results (Figures 5A though E). All long-chain FAs were significantly reduced (all *p*<0.001), particularly 594 (C36H66O6; *p*=5.6E-14, Figure 5A), as were PlsEtns (Figure 5B, all *p*<0.01), lysoPCs (Figure 5C, all *p*<0.001 except 22:4), SMs (Figure 5D, all *p*<0.001), and PtdChos (Figure 5E, all *p*<0.001). To ensure that these results were not artifacts of global phospholipid depletion or lipid breakdown, we assayed several phosphatidylethanolamines (PtdEtns) not identified as significant by FI-FTICR-MS analysis. PtdEtns either showed no change between PC patients and controls, or in some cases, were elevated (Figure 5F). ROCs based on four metabolites, each with the lowest *p*-value for each respective system (594, PtdCho 18:0/18:2, lysoPtdCho 18:2, and sphingomyelin d18:1/24:0), showed AUCs between 0.93 and 0.97 (Figure 5G, 95% confidence intervals shown).

**Figure 6:**
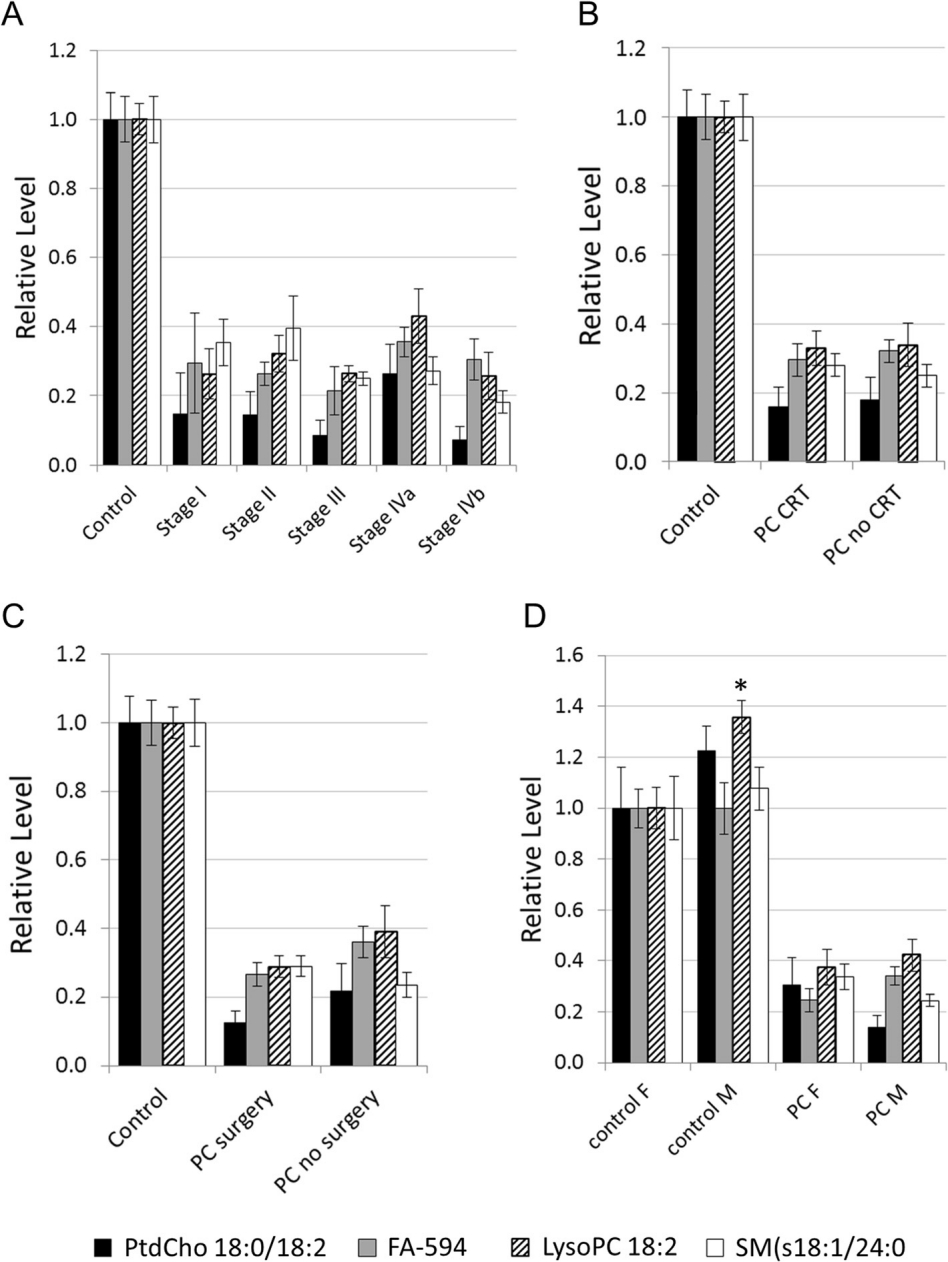
**Effects of stage, treatment, surgery and gender on the four selected metabolites of each system. A**, by disease stage; **B**, by treatment; **C**, by surgery at time of sampling; and **D**, by gender. Results are based on tandem-MS data normalized to control mean, ±1 SEM. Asterisk denotes *p*<0.05 versus female.

We used the same four markers to further investigate potential association with other clinical variables including disease stage, treatment, surgery and gender (Figure 6). No correlations were observed between any of the metabolites and disease stage (Figure 6A), treatment status (Figure 6B), or collection at surgery (Figure 6C). LysoPC18:2 was the only metabolite that showed a slight elevation in males versus females (*p*<0.05, Figure 6D). There were no significant associations between other metabolites of each system and these variables (results not shown).

### Independent population validation

**Figure 7:**
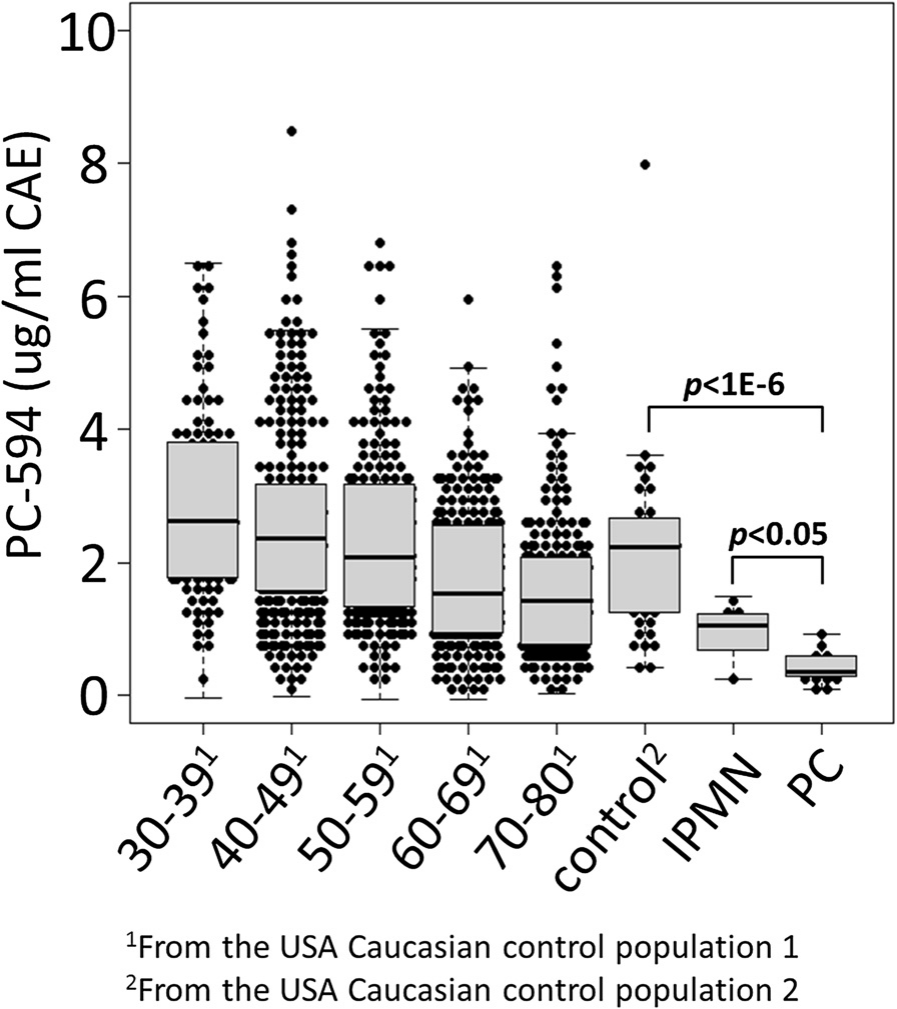
**PC-594 levels among US Caucasians.** Beeswarm jitter plots of PC-594 concentrations (ug/ml CAE ±1 SEM) for 1000 reference subjects by age (USA control population 1), a second control population (n=40, USA control population 2), patients with intraductal papillary mucinous neoplasms (n=6, IPMNs), and PC patients (n=14). Grey boxes represent the 25th to 75^th^ percentile and the whiskers represent the 5th to 95th percentile. Black lines within the grey boxes represent the median.

We determined the distribution of LCFA 594.4862 (PC-594) across a random sampling of 1000 US Caucasian reference subjects (USA control 1) between age 30 and 80 (similar to our previous approach [4]) using a FI-MS/MS quantitative assay based on ^13^C-cholic acid as an internal standard (see Methods). We did this to define a low-risk population based on age (since age is the largest risk factor for PC), and to investigate potential association between PC-594 and age. We then compared the distribution of PC-594 to a second, independent, US Caucasian population of 14 PC patients, six patients with intraductal papillary mucinous neoplasms (IPMNs), and 40 additional confirmed disease-free controls (USA control 2, Figure 7). All samples were blinded prior to analysis.

The mean PC-594 concentration among the 1000 reference subjects was 2.23 ±0.05 ug/ml cholic acid equivalents (CAE), and negatively correlated with age (Figure 7, regression multiple R=0.29, *p*<0.0001). Subjects under age 40 showed a mean level of 2.85 ±0.14 ug/ml CAE, which declined to 1.69 ±0.09 ug/ml CAE for subjects aged 70–80 (Figure 7 and Table 2). The mean concentration of the 40 USA control 2 subjects was 2.15 ±0.2 ug/ml CAE, consistent with that of the USA control group 1 (2.23 ±0.05 ug/ml, *p*=0.7). However, the mean levels of the 14 PC and 6 IPMN patients were 0.43 ±0.06 and 0.96 ±0.19 ug/ml CAE, respectively, representing an approximate five-fold reduction in the circulating levels of PC-594 in PC patients compared to controls.

**Table 2:** PC-594 statistical performance in USA Caucasian populations

**Cohort**	**Mean PC-594 (ug/ml CAE) ±1SEM**	***p *versus PC**	**ROC-AUC versus PC (95% CI)**
**USA control 1**			
30-39 yrs	2.85 ±0.14	4.8E-09	0.99 (0.98-1.0)
40-49 yrs	2.58 ±0.09	5.7E-07	0.97 (0.96-0.98)
50-59 yrs	2.44 ±0.12	1.8E-05	0.97 (0.96-0.98)
60-69 yrs	1.77 ±0.10	1.9E-05	0.91 (0.89-0.93)
70-80 yrs	1.69 ±0.09	7.9E-04	0.90 (0.88-0.92)
All yrs	2.23 ±0.05	6.2E-05	0.94 (0.93-0.96)
**USA control 2**	2.15 ±0.20	5.9E-06	0.97 (0.95-0.99)
**USA IMPN**	0.95 ±0.19	0.003	-
**USA PC**	0.43 ±0.06	-	-

The ROC-AUCs based on PC-594 levels for PC patients versus controls by age are shown in the right column of Table 2. The AUC for PC versus reference subjects aged 30–39 was 0.99 (95% CI 0.98-1.0), with a *p*-value of 4.8E-9. The AUC declined to 0.90 (95% CI 0.88-0.92) by age 70–80, but was still significant with a *p*-value of 7.9E-4. For the general population aged 30–80, the average AUC was 0.94 (95% CI 0.93-0.96). The resulting AUC of PC-594 based on the US control 2 group was 0.97 (95% CI 0.95-0.99). The results showed that even though the AUC declined with increasing age, the discrimination remained high across all ages (>90%). That is, even the oldest reference subjects (aged 70–80) showed PC-594 levels well above those of the PC patients (1.69 versus 0.43, respectively).

We next arbitrarily defined five PC-594 positivity rates between 0.5 and 10% based on low-risk reference subjects (under age 50) and determined the resulting sensitivities and specificities by decade of life (Table 3). For example, PC-594 cut-offs yielding positivity rates of 2.5 and 5% in reference subjects under age 50 resulted in sensitivities of 64% and 87%, respectively. Calibration of predicted versus observed probabilities based upon a 5% PC-594 positivity rate in both USA 1 controls under age 50 and USA2 controls resulted in E_avgs_ of 0.08 and 0.1, and E_maxs_ of 0.12 and 0.14, respectively, indicating reliable predictions (Additional file 4).

**Table 3:** Sensitivities and specificities based on fixed PC-594 positivity rates in low-risk subjects

**% Positivity < age 50 (specificity, 95% CI)**	**USA PC % Sensitivity (95% CI)**	% Specificity* (95% CI)			
		**USA Control 1 by age**			
		**50-59**	**60-69**	**70-80**	**USA control 2**
**0.5** (99.5, 98–100)	7 (0.4-36)	100 (97–100)	99 (96–100)	100 (97–100)	100 (89–100)
**1.0** (99.0, 97–100)	21 (6–52)	99 (95–100)	95 (91–98)	97 (93–99)	100 (89–100)
**2.5** (97.5, 95–99)	64 (36–86)	97 (93–99)	90 (85–94)	93 (88–96)	95 (82–99)
**5.0** (95.0, 92–97)	87 (56–98)	94 (90–97)	84 (78–88)	77 (70–82)	95 (82–99)
**10.0** (90.0, 87–93)	100 (73–100)	90 (85–94)	74 (68–80)	67 (60–73)	83 (67–92)

*assuming no PC among reference subjects.

Despite the reductions in sensitivity and specificity with age, the cut-off correlating with 2.5% positivity in subjects under age 50 resulted in specificities of greater than 90% across all other age groups (Table 3, specificities of 97%, 90% and 93% for ages 50–59, 60–69 and 70–80, respectively), whereas a cut-off resulting in 5.0% positivity resulted in an apparent age-related effect, with specificities of 94%, 84% and 77% for ages 50–59, 60–69 and 70–80, respectively.

### Clinical benefit

We evaluated potential clinical benefit of screening subjects based on PC-594 using decision curve analysis (DCA) [18,19,24]. DCA is based on plotting the net benefit against a threshold probability (*p*_*t*_), or the probability that a patient has the disease. The method is suitable for determining the benefit of incorporating alternative diagnostic methods relative to current practice, independent of test performance criteria such as ROC-AUC, sensitivity and specificity, and study sample size. An advantage of DCA is that it does not require knowledge of all the possible outcomes of clinical decisions typically required for classical decision-analytic methodology [25].

**Figure 8:**
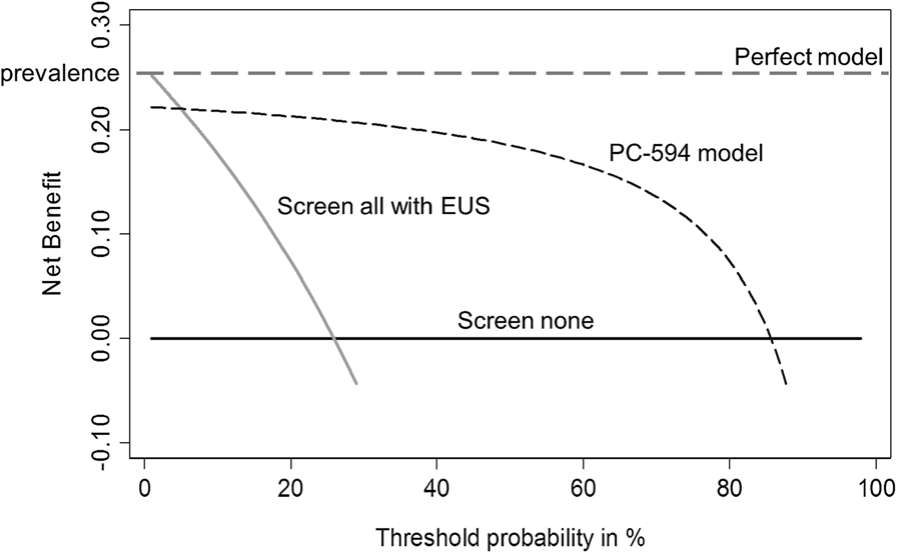
**Decision curve analysis (DCA) for prediction of PC based on PC-594.** The plot compares the net clinical benefits of four scenarios: a perfect prediction model (grey dashed line), screen none (solid horizontal black line), screen everyone with EUS (grey line), and screen based on the PC-594 model. Data for the plot was based on the USA2 validation cohort (PC prevalence of 26%). See Results for further explanation.

We used DCA to determine whether prescreening subjects with PC-594 would provide a net benefit for patients over sending all subjects for EUS (or treatment in our case), versus performing no screening of any kind (the current paradigm for PC). Let $\hat{p}$ be the probability of having PC and *p*_*t*_ the probability of having the disease. *p*_*t*_ represents the probability for which a doctor or patient considers the risk sufficient to warrant further treatment. Generally a patient will choose treatment if $\hat{p}$ >*p*_*t*_. The resulting decision curve, shown in Figure 8, compares the net benefits of screening with PC-594 at various *p*_*t*_’s versus treating all (everyone undergoes EUS), and treating none. The net benefit (y-axis) can be interpreted as the additional percent of true positives that would be detected without an increase in the number of false positives (or in our case, patients required to undergo EUS who don’t have cancer). A perfect model would result in a net benefit of identifying all patients (equal to the prevalence) regardless of the *p*_*t*_.

DCA based on our data (Figure 8) showed substantial net clinical benefit with PC-594 for all *p*_*t*_’s above 10%. Using 20% *p*_*t*_ as an example (i.e. a patient feels that a 20% probability of having PC is sufficient risk to warrant EUS), the net benefit is 0.14 greater than for performing EUS on all subjects (0.22-0.08). This translates into the finding of 14/100 additional cases tested without an increase in unnecessary procedures, compared to scoping all subjects. This represents a substantial net clinical benefit, particularly in the case of a disease that currently lacks any screening modalities.

## Discussion

To date, carbohydrate antigen 19.9 (CA-19.9) is the only biomarker routinely used, and FDA approved, for the clinical management of PC. However, its primary uses are for prognosis [26-28] and for monitoring high-risk populations [29]. CA-19.9 has questionable value for average-risk screening [28,30], and due to low sensitivity and specificity, the American Society of Clinical Oncology (ASCO) does not recommend the use of CA19.9 for diagnostic screening purposes regardless of symptoms [31]. In the discovery PC patient population reported herein, only 21 of the 40 patients had CA19.9 levels greater than 35 U/ml (52.5% sensitivity; results not shown), consistent with the abovementioned reports. Accordingly, there are currently no viable means to screen for increased risk of, or early-stage PC.

The non-targeted metabolomics discovery platform used in this study has previously identified early-stage biomarkers mechanistically involved in Alzheimer’s disease [16], autism [32], and colorectal cancer [15]. The key advantages of this platform are that: 1) Samples are processed using a liquid-liquid extraction followed by direct infusion of each extract without chromatography where all molecules are introduced into the system and can therefore potentially be detected. 2) The ultra-high resolution of the FTICR-MS enables mass measurements with accuracy sufficient for the computational determination of elemental composition, and rapid insight into the identities of peaks. 3) Translation of FI-FTICR-MS discoveries into sensitive and cost-effective targeted and quantitative FI-MS/MS assays is seamless due to the high compatibility of the two systems. The high correlation between the non-targeted FI-FTICR-MS and targeted FI-MS/MS results shown in this study validates high-resolution non-targeted metabolomics as a highly sensitive and accurate tool for *de novo* biomarker discovery applications.

Overall, the difference in magnitude between the PC patient and control subject serum metabolomes we observed in this study was remarkable. In fact, PCA performed on the entire dataset of 2478 masses, with no prior filtering, clearly discriminated PC patients from controls (not shown). This was due to multiple affected metabolic systems each containing numerous similarly-behaving components. By clustering the FI-FTICR-MS data by Pearson correlation, it was possible to quickly identify these systems. However, the limitation of FI-FTICR-MS, particularly for intact glycerolipids, is that the fatty acid side-chain speciations can only be speculated due to isomerism. For example, differentiating 16:0/18:3 at SN1/SN2 from 16:1/18:2 is not possible. FI-MS/MS therefore represents an ideal complimentary approach because it not only allows for the confirmation of side-chain speciations, but also the investigation of related metabolites that may not have been detected with FI-FTICR-MS due to low abundance, etc.

Using this two-pronged approach, we discovered and confirmed the involvement of three major dysregulated metabolic systems in the serum of PC patients: ultra long-chain fatty acids, numerous choline-containing glycerophospholipids, and vinyl ether-containing ethanolamine phospholipids called plasmalogens. Although most of the individual metabolites alone showed a significant reduction in PC patient serum, the strongest discriminator based on multiple statistical criteria was PC-594 (*p*=9.9E-14).

The ROC-AUCs based on PC-594 were highly consistent, independent of the platform used or population evaluated in this study (0.98 based on FI-FTICR-MS analysis of the discovery samples, 0.96 based on FI-MS/MS confirmation of the discovery samples, and 0.97 based on the blinded analysis of the US Caucasian patient cohort). On average, the mean PC-594 concentration in PC patients was more than five times lower than control subjects. PC-594 reduction was not observed to correlate with either the magnitude of disease burden (as assessed by stage) or treatment. Interestingly, PC-594 levels were also reduced in IPMN patients. Although IPMN is technically a cystic tumor, it is still a cancer with an invasive component in a high percentage of cases [33]. These results suggest the possibility that the tumor is not responsible for the reduction, but rather that the reduction precedes the onset of disease, similar to GTA-446 reduction and CRC [4,34].

Because the RF predictor based on 11 masses (Figure 4A), showed little improvement in diagnostic accuracy over PC-594 alone (the ROC-AUC based solely on PC-594 was 0.96 versus 0.98 for the RF model), we proceeded with further validation of PC-594 only. A single-analyte assay has several advantages including simpler method development, quantitation, and an easier regulatory approval path. However, our results also showed a strong association between phosphocholine reduction and PC, and therefore evaluating subjects for choline-related deficiency as an independent risk factor should not be disregarded.

Before commenting on the biological implications of the findings, two key limitations of the study should be addressed. First, the current study did not include subjects with non-malignant pancreatic-related conditions such as pancreatitis or jaundice. Since a high percentage of PC patients exhibit jaundice, and up to 5% of subjects with pancreatitis develop PC in a 20-year period [35], we cannot exclude the possibility that these conditions are also linked to the metabolic effects observed. Although subjects with these conditions would not necessarily be representative of a low-risk target screening population, it will be important to determine whether these conditions affect the biomarkers reported herein, particularly in light of recent reports that jaundice can impact performance of certain PC protein markers [36].

Second, the sample sizes of the studies were not large. In particular, the clinical diversity of the discovery population was high, including samples from patients collected at time of surgery, following treatment, as well as a low number of cases by disease stage. Although we observed no bias toward any of these variables, (Figures 1B and 6), interpretation (especially for the lack of disease stage effect) should be taken with caution.

The biological implications of reduced systemic levels of long-chain FAs and numerous classes of glycerophospholipids in PC patients are intriguing and warrant further discussion. The long-chain FAs, although only recently reported, represent a large family of 28 to 36 carbon polyhydroxylated and polyunsaturated long-chain fatty acids, originally named gastric-tract acids (GTAs) for their role in CRC [15]. The prototypical member of the family, GTA-446, has 28 carbons and is reduced in colorectal cancer patients relative to control subjects [4,15,34].

In previous studies, we showed that human serum extracts enriched for selected GTAs protected against inflammation through the down-regulation of NFκB and several pro-inflammatory markers in both human colon cancer and RAW264.3 mouse macrophage cells exposed to lipopolysaccharide [37]. GTA-treated cells also showed reduced proliferative capacity through a pro-apoptotic mechanism [37].

In colon cancer, the current hypothesis is that GTAs act analogously to the resolvins and protectins [38], protecting the body against the accumulation of chronic inflammation over time. Compromised levels with age are suspected to favor the establishment of a pro-inflammatory environment, and ultimately lead to the DNA damage observed in many tumors. PC-594 belongs to the same metabolic system as GTA-446; therefore, it is probable that PC-594, at least to some extent, is also involved in inflammatory processes. Given that PC incidence is low in subjects under age 45 (<3% of cases) and increases with age thereafter (SEER data, 2005–2009), it is tempting to speculate whether the age-related reduction of PC-594 could be causally involved in the establishment of PC.

Given the role of GTAs in inflammation, our current work is focused on determining whether subjects with chronic pancreatitis, an inflammatory condition, have altered levels of PC-594 and other ultra long-chain FAs. Likewise, further investigation of GTA family members across different cancers and inflammatory conditions is warranted for dissecting the specific roles that different isoforms play in the causation of these diseases.

Phosphocholine metabolism has also been previously implicated with PC. For example, results by Yao *et al.* showed decreased choline levels in PC tumors via proton MR [39], and others have shown that human cancer cell growth, including PC cells, can be inhibited by various sphingolipids [40,41]. Fang *et al.* showed by NMR that rats with PC exhibited lower phosphocholine and glycerophosphocholine compared to rats with chronic pancreatitis [42]. One of the most convincing *in vivo* studies to date functionally implicating choline metabolism to PC was by Longnecker *et al.*, who showed that rats fed a choline-supplemented diet exhibited significantly reduced PC lesion areas, lesion diameters and numbers of lesions compared to rats fed a choline-devoid diet. This lead the authors to conclude that a choline-deficient diet might have a growth promoting activity [43].

Choline is also important for pancreatic cell function, as pancreatic acinar cell integrity and the generation of digestive enzymes and insulin secretion are dependent upon high choline phospholipid metabolism [44]. There is also evidence that reduced sphingomyelin levels may be oncogenic as demonstrated by inhibition of the RAS-MAPK, CyclinD-CDK4/CDK6 and PI3K-AKT axes through the activation of sphingomyelin synthase by a synthetic fatty acid [45]. Our finding of reduced circulating levels of choline-based metabolites as possible contributing factors to the development of PC is consistent with these observations.

Our observation of reduced PlsEtns (containing the signature vinyl-ether bond at the SN1 position), but not their diacy counterparts, is also intriguing for several reasons. PlsEtns are membrane phospholipids, primarily located in cells of the nervous system and heart [46,47], but which are produced exclusively by peroxisomes in the liver [48]. The vinyl-ether bond at the SN1 position is required for the lipid’s anti-oxidant role and its effect on membrane fluidity, which is relevant to neuronal impairment because it affects vesicular fusion (for review see [49,50]). We previously reported that reduced systemic levels of PlsEtns in Alzheimer’s disease patients correlate with levels in the brain and cognitive parameters [16]. PlsEtns, however, have also been implicated in cancer [51,52], and plasmalogen analogues have been shown to exhibit anti-tumor properties [53]. The consequence of decreased PlsEtn levels, via effects on membrane structure and microdomain architecture, could impact growth factor receptor-mediated signaling.

The findings of this study represent an opportunity for identifying subjects with PC or a high-risk of developing PC. Consider, for example, current CRC screening guidelines, which suggest that the benefit of identifying early-stage CRC (i.e. the increase in 5-year survival) via endoscopic examination in an asymptomatic population with an incidence rate of 0.05% [54] outweighs the combined risks of complications from endoscopy and late-stage detection mortality. Given the current PC incidence rate of approximately 9.5/100,000 (0.0095%), a blood test with 95% specificity and sensitivity approaching 90% would yield a PC detection rate of approximately 8.6 per 5000 positive tests, or 0.17%. This represents an 18-fold increase in PC risk over average-risk subjects given a positive PC-594 test, and a 3-fold higher incidence rate than the current incidence considered sufficient for colonoscopy-based screening of CRC in the general population. Considering the high mortality rate of late-stage detection, the benefits of endoscopic screening in a small population of high-risk subjects with low PC-594 levels becomes obvious.

Improved clinical benefit was further supported by the results of decision curve analysis, which showed significant net clinical benefit above all *p*_*t*_’s greater than 10% compared to screening all subjects with EUS, which was not surprising given the high discriminating ability of PC-594. The results of our studies clearly implicate a circulating reduction of PC-594 in PC, and establish the foundation for designing future prospective trials to determine the net clinical benefit of PC-594 screening in the true average-risk population.

## Conclusions

Using a sensitive, high-resolution mass spectrometry-based platform, we showed that the serum metabolome of PC patients is significantly altered. We confirmed the findings using independent populations and triple-quadrupole tandem mass spectrometry assays designed for specific metabolic systems. Specifically, PC patients showed severely compromised levels of several classes of serum phospholipids and novel ultra long-chain fatty acids. In particular, fatty acid PC-594 showed an AUC of greater than .95 for discriminating PC patients from controls in two geographically and ethnically diverse populations. The findings are relevant in the context of screening because the enrichment of PC in PC-594 deficient populations suggests that reducing PC mortality rates via screening and early-detection is plausible. The design and implementation of a suitable clinical trial to test this hypothesis is now underway.

## Abbreviations

APCI: Atmospheric pressure chemical ionization; AUC: Area under the curve; BuOH: Butanol; CA-19.9: Carbohydrate antigen 19.9; CAE: Cholic acid equivalent; CID: Collision induced dissociation; DCA: Decision curve analysis; ESI: Electrospray ionization; EtOAc: Ethyl acetate; FA: Fatty acid; FDR: False discovery rate; FI-FTICR-MS: Flow-injection fourier transform ion cyclotron resonance mass spectrometry; FI-MS/MS: Flow injection tandem mass spectrometry; GTA: Gastric-tract acid; IPMN: Intraductal papillary mucinous neoplasms; lysoPC: Lysophosphatidylcholine; MRM: Multiple reaction monitoring; PC: Pancreatic cancer; PCA: Principal component analysis; PlsEtn: Plasmalogen ethanolamine; PtdCho: Phosphatidylcholine; PtdEtn: Phosphatidylethanolamine; RF: Random forest; ROC: Receiver-operator characteristic; SM: Sphingomyelin.

## Competing interests

Shawn Ritchie, Wei Jin, Elodie Pastural, Tolulope Sajobe, Dushmanthi Jayasinghe, Bassirou Chitou and Yasuyo Yamazaki were paid employees of Phenomenome Discoveries, Inc. Dayan Goodenowe is a director of Phenomenome Discoveries, Inc. The other authors of have no competing interests. Phenomenome Discoveries, Inc. financed the publication costs of this paper. Part of the results discussed in this paper have been provisionally filed for patent (U.S. application number 13/499,369).

## Authors’ contributions

SAR, HA, IT, YY and DBG designed the metabolomic studies. SAR and DBG were the primary authors. IT, HE, HN, MM, YD and MM were responsible for the Japanese trial design, patient enrollment, clinical data management and interpretation of findings. SAR and EP performed multivariate statistics and analysis of FI-FTICR-MS and FI-TQ-MS/MS data. BC and TTS performed the Random Forest, jitter plots, ROC and clinical performance statistical analyses. TTS performed the calibration and decision curve analysis. DJ and WJ developed the FI-TQ-MS/MS assays. TW was responsible for the USA Caucasian study design and blinded analysis. All authors had input, read, and agree with the contents of the manuscript. All authors read and approved the final manuscript.

## Pre-publication history

The pre-publication history for this paper can be accessed here:

http://www.biomedcentral.com/1471-2407/13/416/prepub

## Supplementary Material

Additional file 1 **Transition lists for tandem-MS methods.** The file lists the parent-daughter ion transitions for the ultra long-chain fatty acid system (**Table S1**), the plasmenylethanolamine (plasmalogen) system (**Table S2**), the lysophosphatidylcholine system (**Table S3**), the sphingomyelin system (**Table S4**), the phosphatidylcholine system (**Table S5**) and the phosphatidylethanolamine system (**Table S6**). Click here for file

Additional file 2 **PCA parameters.** The file lists the proportional and cumulative variance by factor and the chi2 results (“PCA variance” tab), the square factor loadings (Q2) for each mass across each factor the and square multiple correlation (R2) for each mass (“PCA Q2R2” tab), the loadings for each mass across each factor and the uniqueness for each mass (“loadings uniqueness” tab), and the *p*-value of each mass based on pairwise comparisons between PC and controls, females and males, stages of disease (within PC), treatment status (within PC) and surgery (within PC) (“p-value for bias” tab). Click here for file

Additional file 3 **Random Forest output parameters.** A). The RF classification accuracy plot, and B). the Gini index for masses used in the training phase. C), Calibration plot based on the predicted probabilities of the test-set data. Click here for file

Additional file 4 **Calibration plots.** Calibration of the model to predict PC based on PC-594. The x-axis represents the predicted probability of PC and the y-axis represents the actual probability of PC. A) calibration plot for PC versus USA2 controls, B) calibration plot for PC versus USA1 controls < age 50. The average differences (E_avg_) and maximum differences (E_max_) between the predicted model and a perfect model (the straight diagonal), are shown on each plot. Click here for file

Additional file 5 **Representative tandem-MS spectra and selected ion currents of selected metabolites.** The CID-induced fragmentation pattern of a representative member from each of the four metabolic systems is shown along the left. Arrows indicate the daughter ions used for quantitation. The selected ion currents for each of the daughter ions are shown for five randomly-selected normal and five pancreatic patient samples (right side). See Methods and Additional file 1 for more information. Click here for file
