# Supplementary material for: Metabolic system alterations in pancreatic cancer patient serum: potential for early detection
Source: BMC Cancer. 2013 Sep 12;13:416. doi: 10.1186/1471-2407-13-416 (PMC3847543; doi:10.1186/1471-2407-13-416)
Supplement: Additional file 1 — Transition lists for tandem-MS methods. The file lists the parent-daughter ion transitions for the ultra long-chain fatty acid system (Table S1), the plasmenylethanolamine (plasmalogen) system (Table S2), the lysophosphatidylcholine system (Table S3), the sphingomyelin system (Table S4), the phosphatidylcholine system (Table S5) and the phosphatidylethanolamine system (Table S6). [file 1471-2407-13-416-S1.docx]

**Additional file 1.**

Table 1: Ultra long-chain fatty acid system tandem-MS panel:

| **Metabolite Name** | **Molecular Formula** | **Parent Mass (neutral)** | **MRM Transitions Monitored (M-H)** |
| --- | --- | --- | --- |
| GTA-558 | C36H62O4 | 558.4 | 557.4 / 495.4 |
| GTA-574 | C36H62O5 | 574.5 | 573.5 / 511.4 |
| GTA-576 | C36H64O5 | 576.5 | 575.5 / 513.5 |
| GTA-578 | C36H66O5 | 578.5 | 577.5 / 515.4 |
| GTA-592 | C36H64O6 | 592.5 | 591.5 / 555.4 |
| GTA-594 | C36H66O6 | 594.5 | 593.5 / 557.5 |
| GTA-596 | C36H68O6 | 596.5 | 595.5 / 559.5 |

Table 2: Plasmenylethanolamine system tandem-MS panel:

| **Metabolite Name** | **Molecular Formula** | **Parent Mass (neutral)** | **MRM Transitions Monitored (M-H)** |
| --- | --- | --- | --- |
| PlsEtn 16:0/18:1 | C39H76NO7P | 701.5 | 700.5 / 281.2 |
| PlsEtn 16:0/18:2 | C39H74NO7P | 699.5 | 698.5 / 279.2 |
| PlsEtn 16:0/18:3 | C39H72NO7P | 697.5 | 696.5 / 277.2 |
| PlsEtn 16:0/20:4 | C41H74NO7P | 723.5 | 722.5 / 303.2 |
| PlsEtn 16:0/22:6 | C43H74NO7P | 747.5 | 746.5 / 327.2 |
| PlsEtn 18:0/18:1 | C41H80NO7P | 729.5 | 728.5 / 281.2 |
| PlsEtn 18:0/18:2 | C41H78NO7P | 727.5 | 726.5 / 279.2 |
| PlsEtn 18:0/18:3 | C41H76NO7P | 725.5 | 724.5 / 277.2 |
| PlsEtn 18:0/20:4 | C43H78NO7P | 751.5 | 750.6 / 303.2 |
| PlsEtn 18:0/22:6 | C45H78NO7P | 775.5 | 774.5 / 327.2 |
| PlsEtn 18:1/18:1 | C41H78NO7P | 727.5 | 726.5 / 281.2 |
| PlsEtn 18:1/18:2 | C41H76N1O7P1 | 725.5 | 724.5 / 279.2 |
| PlsEtn 18:1/18:3 | C41H74N1O7P1 | 723.5 | 722.5 / 277.2 |
| PlsEtn 18:1/20:4 | C43H76N1O7P1 | 749.5 | 748.5 / 303.2 |
| PlsEtn 18:1/22:6 | C45H76N1O7P1 | 773.5 | 772.5 / 327.2 |
| Free 22:6 | C22H32O2 | 328.2 | 327.2 / 283.2 |
| Free 20:4 | C20H32O2 | 304.2 | 303.2 / 259.2 |

Table 3: Lysophosphatidylcholine system tandem-MS panel:

| **Metabolite Name** | **Molecular Formula** | **Parent Mass (neutral)** | **Formic Acid Adduct (M-H)** | **MRM Transitions Monitored (M-H)** |
| --- | --- | --- | --- | --- |
| LysoPC 14:0 | C22H46NO7P | 467.3 | 512.3 | 512.3 / 227.2 |
| LysoPC 16:0 | C24H50NO7P | 495.3 | 540.3 | 540.3 / 255.2 |
| LysoPC 16:1 | C24H48NO7P | 493.3 | 538.3 | 538.3 / 253.3 |
| LysoPC 18:0 | C26H54NO7P | 523.4 | 568.4 | 568.4 / 283.3 |
| LysoPC 18:1 | C26H52NO7P | 521.3 | 566.3 | 566.3 / 281.3 |
| LysoPC 18:2 | C26H50NO7P | 519.3 | 564.3 | 564.3 / 279.3 |
| LysoPC 18:3 | C26H48NO7P | 517.3 | 562.3 | 562.3 / 277.3 |
| LysoPC 20:1 | C28H56NO7P | 549.4 | 594.4 | 594.4 / 309.3 |
| LysoPC 20:2 | C28H54NO7P | 547.4 | 592.2 | 592.4 / 307.3 |
| LysoPC 20:3 | C28H52NO7P | 545.4 | 590.3 | 590.3 / 305.2 |
| LysoPC 20:4 | C28H50NO7P | 543.3 | 588.3 | 588.3 / 303.2 |
| LysoPC 20:5 | C28H48NO7P | 541.3 | 586.3 | 586.3 / 301.2 |
| LysoPC 20:6 | C28H46NO7P | 539.3 | 584.3 | 584.3 / 299.2 |
| LysoPC 22:5 | C30H52NO7P | 569.3 | 614.3 | 614.3 / 329.2 |
| LysoPC 22:6 | C30H50NO7P | 567.3 | 612.3 | 612.3 / 327.2 |

Table 4: Sphingomyelin tandem-MS panel.

| **Metabolite Name** | **Molecular Formula** | **Parent Mass (neutral)** | **Formic Acid Adduct (M-H)** | **MRM Transition 1 Monitored (M-H)** | **MRM Transition 2 Monitored (M-H)** |
| --- | --- | --- | --- | --- | --- |
| SM(d18:1/24:1) | C47H93N2O6P | 812.6 | 857.6 | 857.6 / 797.6 | 857.6 / 168.1 |
| SM(d18:1/24:0) | C47H95N2O6P | 814.6 | 859.6 | 859.6 / 799.6 | 859.6 / 168.1 |
| SM(d18:1/18:1) | C41H81N2O6P | 728.6 | 773.6 | 773.6 / 713.6 | 773.6 / 168.1 |
| SM(d18:1/18:0) | C41H83N2O6P | 730.6 | 775.6 | 775.6 / 715.6 | 775.6 / 168.1 |
| SM(d18:1/16:0) | C39H79N2O6P | 702.6 | 747.6 | 747.6 / 687.6 | 747.6 / 168.1 |

Table 5: Phosphatidylcholine tandem-MS panel:

| **Metabolite Name** | **Molecular Formula** | **Parent Mass (neutral)** | **Formic Acid Adduct (M-H)** | **Qualifier** | **MRM Transitions Monitored (M-H)** |
| --- | --- | --- | --- | --- | --- |
| PtdCho 16:0/18:0 | C42H84NO8P | 761.6 | 806.6 | 746.6 | 806.6 / 744.6 / 255.2 |
| PtdCho 16:0/18:1 | C42H82NO8P | 759.6 | 804.6 | 744.6 | 804.6 / 744.6 / 281.2 |
| PtdCho 16:0/18:2 | C42H80NO8P | 757.6 | 802.6 | 742.6 | 802.6 / 742.6 / 279.2 |
| PtdCho 16:0/18:3 | C42H78NO8P | 755.6 | 800.6 | 740.6 | 800.6 / 740.6 / 277.6 |
| PtdCho 16:0/20:3 | C44H82NO8P | 783.6 | 828.6 | 768.6 | 828.6 / 768.6 / 305.3 |
| PtdCho 16:0/20:4 | C44H80NO8P | 781.6 | 826.6 | 766.6 | 826.6 / 766.6 / 303.2 |
| PtdCho 16:0/20:5 | C44H78NO8P | 779.6 | 824.6 | 764.6 | 824.6 / 764.6 / 301.2 |
| PtdCho 16:1/18:1 | C42H80NO8P | 757.6 | 802.6 | 742.6 | 802.6 / 742.6 / 281.2 |
| PtdCho 16:1/18:2 | C42H78NO8P | 755.6 | 800.6 | 740.6 | 800.6 / 740.6 / 279.2 |
| PtdCho 18:0/18:2 | C44H84NO8P | 785.6 | 830.6 | 770.6 | 830.6 / 770.6 / 279.2 |
| PtdCho 18:1/18:2 | C44H82NO8P | 783.6 | 828.6 | 768.6 | 828.6 / 768.6 / 279.2 |
| PtdCho 18:2/18:3 | C44H78NO8P | 779.6 | 824.6 | 764.6 | 824.6 / 764.6 / 279.2 |

Table 6: Phosphatidylethanolamine system tandem-MS panel:

| **Metabolite Name** | **Molecular Formula** | **Parent Mass (neutral)** | **MRM Transitions Monitored (M-H)** |
| --- | --- | --- | --- |
| PtdEtn 16:0/18:1 | C39H76N1O8P1 | 717.53083 | 716.5 / 255.2 |
| PtdEtn 16:0/18:2 | C39H74N1O8P1 | 715.51518 | 714.5 / 255.2 |
| PtdEtn 16:0/18:3 | C39H72N1O8P1 | 713.49953 | 712.5 / 255.2 |
| PtdEtn 16:0/20:4 | C41H74N1O8P1 | 739.51518 | 738.5 / 255.2 |
| PtdEtn 16:0/22:6 | C43H74N1O8P1 | 763.51518 | 762.5 / 255.2 |
| PtdEtn 18:0/18:1 | C41H80N1O8P1 | 745.56213 | 744.5 / 283.2 |
| PtdEtn 18:0/18:2 | C41H78N1O8P1 | 743.54648 | 742.5 / 283.2 |
| PtdEtn 18:0/18:3 | C41H76N1O8P1 | 741.53083 | 740.5 / 283.2 |
| PtdEtn 18:0/20:4 | C43H78N1O8P1 | 767.54648 | 766.5 / 283.2 |
| PtdEtn 18:0/22:6 | C45H78N1O8P1 | 791.54648 | 790.5 / 283.2 |
| PtdEtn 18:1/18:1 | C41H78N1O8P1 | 743.54651 | 742.5 / 281.2 |
| PtdEtn 18:1/18:2 | C41H76N1O8P1 | 741.53086 | 740.5 / 281.2 |
| PtdEtn 18:1/18:3 | C41H74N1O8P1 | 739.51521 | 738.5 / 281.2 |
| PtdEtn 18:1/20:4 | C43H76N1O8P1 | 765.53086 | 764.5 / 281.2 |
| PtdEtn 18:1/22:6 | C45H76N1O8P1 | 789.53086 | 788.5 / 281.2 |
