# Supplementary figures and images for: Metabolic system alterations in pancreatic cancer patient serum: potential for early detection
Source: BMC Cancer. 2013 Sep 12;13:416. doi: 10.1186/1471-2407-13-416 (PMC3847543; doi:10.1186/1471-2407-13-416)

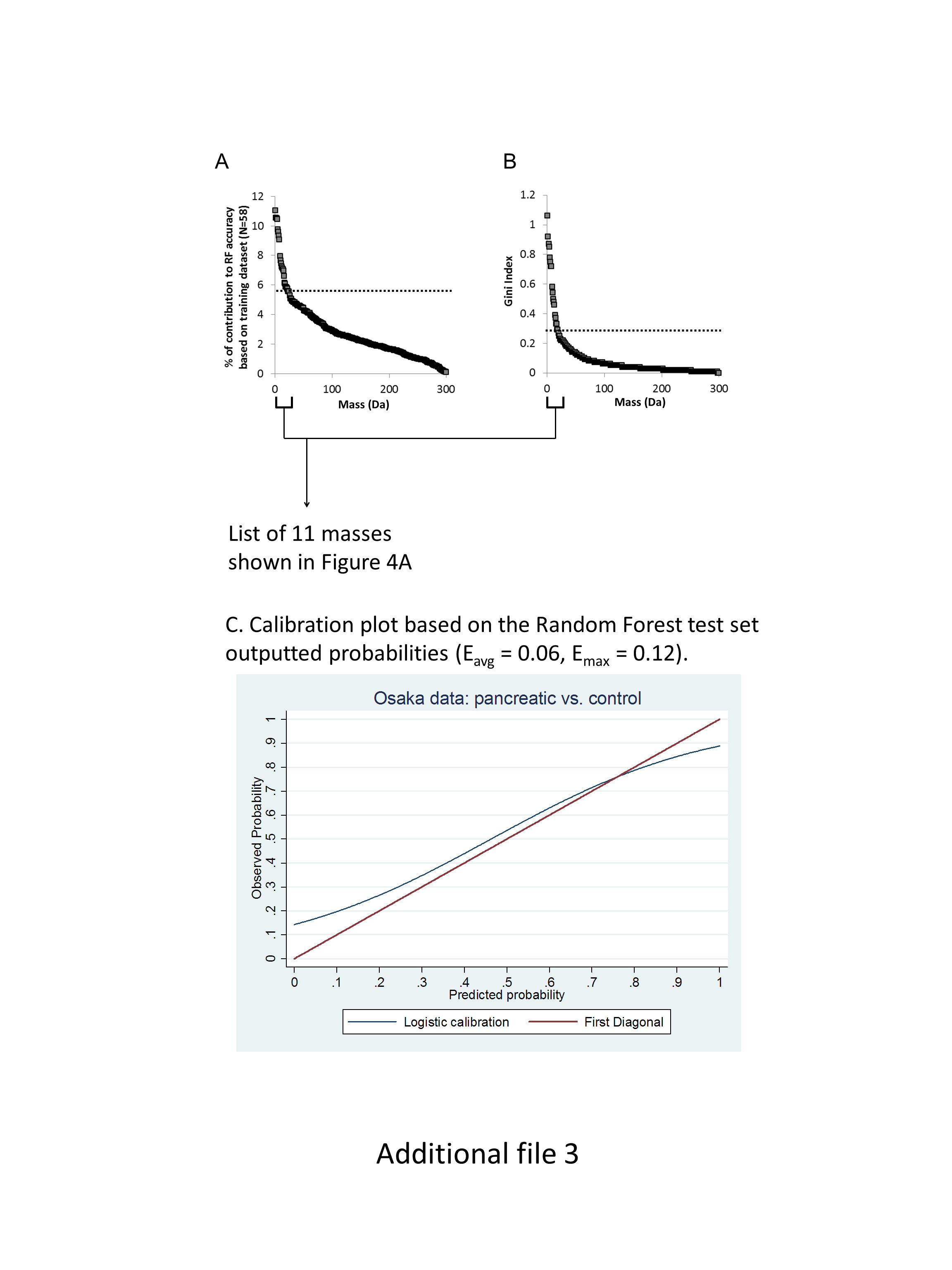

Supplement: Additional file 3 — Random Forest output parameters. A). The RF classification accuracy plot, and B). the Gini index for masses used in the training phase. C), Calibration plot based on the predicted probabilities of the test-set data. [file 1471-2407-13-416-S3.tiff]

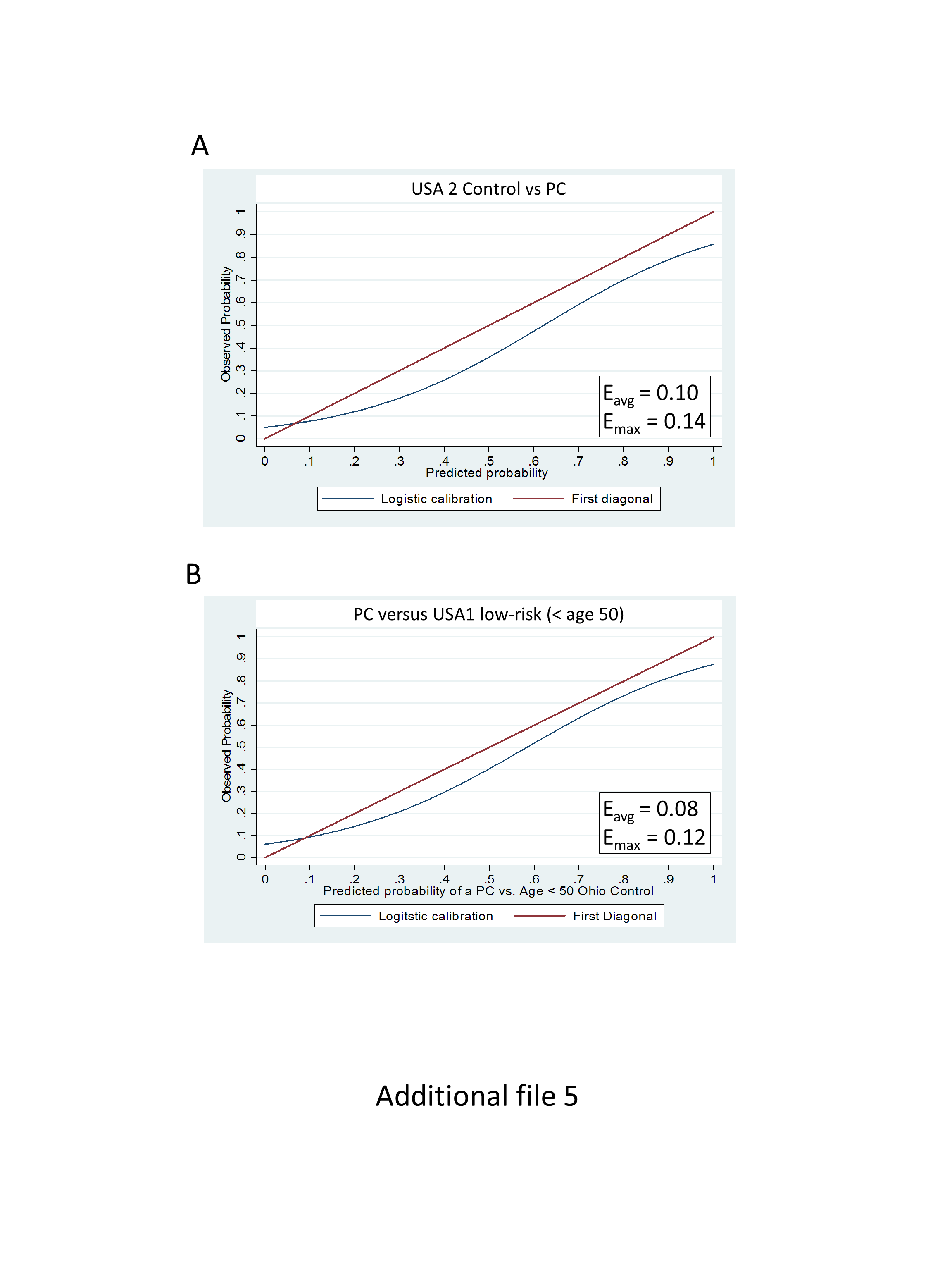

Supplement: Additional file 4 — Calibration plots. Calibration of the model to predict PC based on PC-594. The x-axis represents the predicted probability of PC and the y-axis represents the actual probability of PC. A) calibration plot for PC versus USA2 controls, B) calibration plot for PC versus USA1 controls < age 50. The average differences (Eavg) and maximum differences (Emax) between the predicted model and a perfect model (the straight diagonal), are shown on each plot. [file 1471-2407-13-416-S4.tiff]

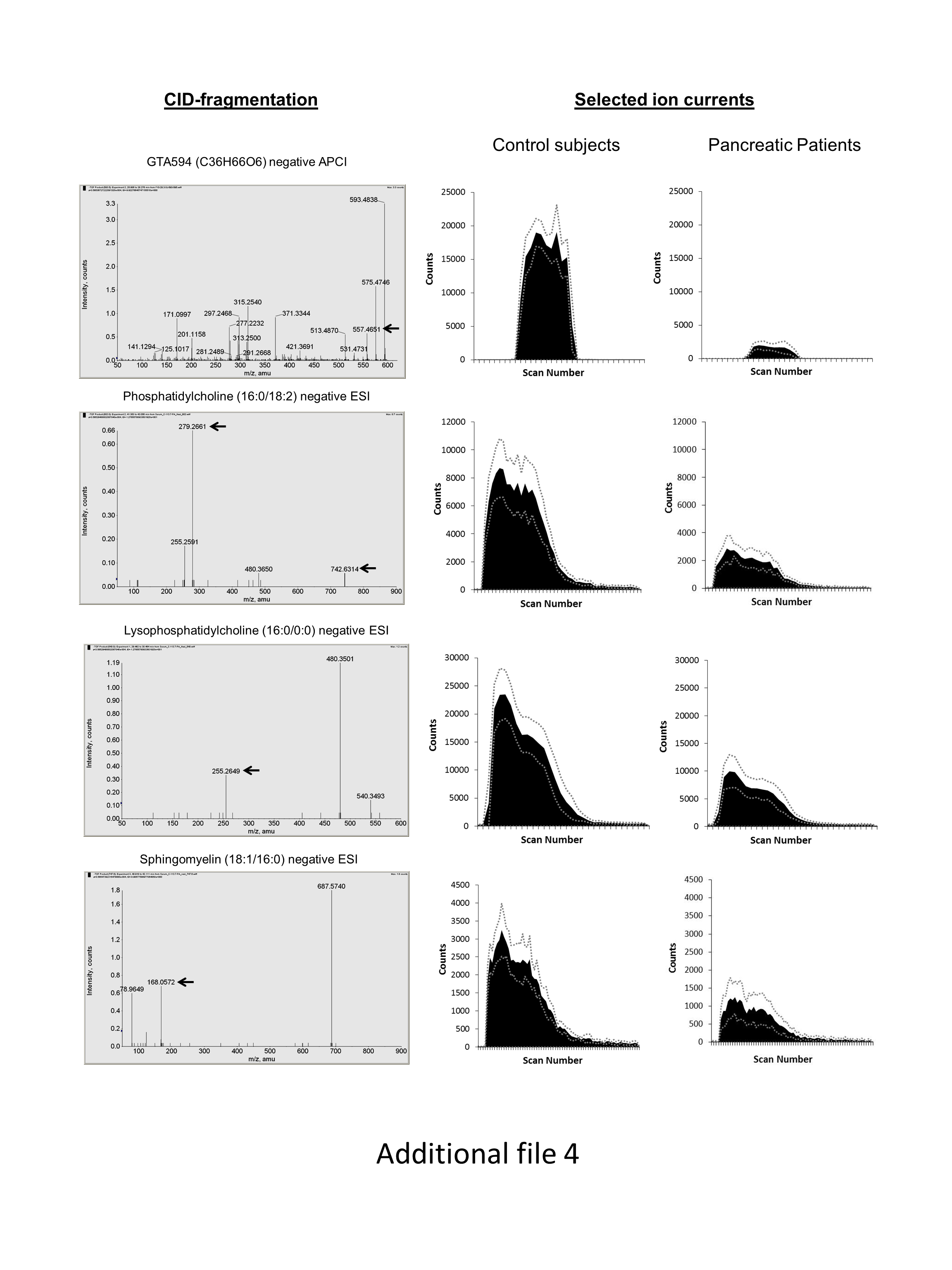

Supplement: Additional file 5 — Representative tandem-MS spectra and selected ion currents of selected metabolites. The CID-induced fragmentation pattern of a representative member from each of the four metabolic systems is shown along the left. Arrows indicate the daughter ions used for quantitation. The selected ion currents for each of the daughter ions are shown for five randomly-selected normal and five pancreatic patient samples (right side). See Methods and Additional file 1 for more information. [file 1471-2407-13-416-S5.tiff]
